# The effect of septal basal segments in the assessment of systolic dyssynchrony index

**DOI:** 10.1186/1532-429X-15-S1-P77

**Published:** 2013-01-30

**Authors:** Avan Suinesiaputra, David A Bluemke, Brett R Cowan, Pau Medrano-Gracia, Joao A Lima, Perry E Radau, Alistair A Young

**Affiliations:** 1 Anatomy with Radiology, University of Auckland, Auckland, New Zealand; 2 National Institute of Biomedical Imaging and Bioengineering, NIH Clinical Center, Bethesda, MD, USA; 3 Cardiovacular Imaging, John Hopkins University, Baltimore, MD, USA; 4 Sunnybrook Research Institute, Sunnybrook Health Sciences Centre, Toronto, ON, Canada

## Background

LV intraventricular dyssynchrony is commonly assessed by systolic dyssynchrony index (SDI), which is defined by the dispersion of time of minimum regional cavity volume. Several studies have reported conflicting results on cardiac resynchronization therapy (CRT) responses based on SDI values. In this study, we aim to characterise SDI distributions in asymptomatic patients and to analyse how particular regions affect the global SDI measurement in different patient groups.

## Methods

We randomly selected 292 asymptomatic patients (EF:60.9±5.9%, LVM:135.2±36.2gr) from the MESA cohort [1], and 36 patients from the Sunnybrook Cardiac dataset [2]: 12 patients with hypertrophy (EF:63.0±3.7%, LVM:176.1±86.2gr) and 24 heart failure subjects (EF:33.6±12.3%, LVM:197.1±41.0gr). Three-dimensional LV models were semi-automatically generated using dedicated software (CIM v6.0, AMRG, Auckland) from cine MRI. To register and normalise the temporal domain (0≤t≤1), LV models between frames were interpolated using smooth periodic spline method. ED frames were registered at t=0. The AHA 17-segment models were determined at ED and then propagated to all frames to get consistent regions. Segment 17 was excluded. SDI was computed by taking the standard deviation of normalised time to reach peak temporal ejection fraction (EF) from all regions.

## Results

**Figure 1:**
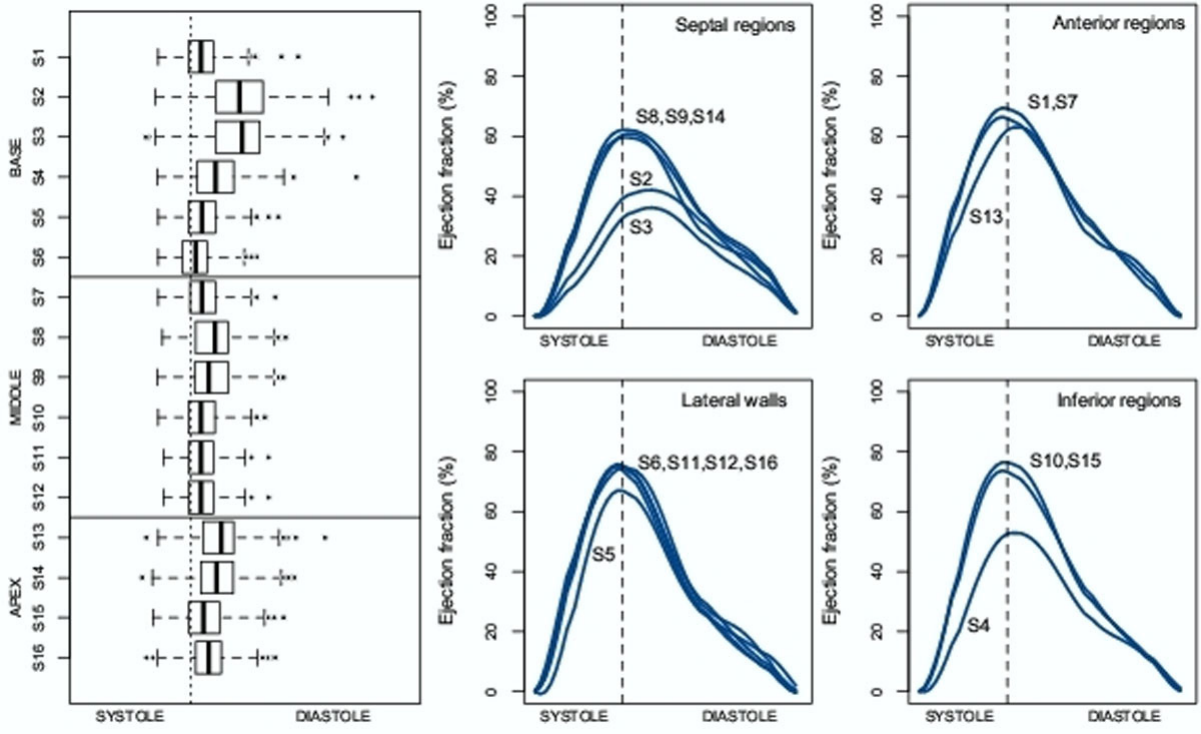
Left: The distributions of the peak time of regional EF values. Right (four figures): temporal profiles of the average regional EF values grouped in four regions of myocardium (septum, anterior, lateral and inferior). All of these plots were computed from the asymptomatic patient group (n=292).

The distributions of regional EF in asymptomatic patients in Fig. 1 (left) suggest a wider range of peak time and a significant median shift to late systole in two basal septal regions (S2 and S3). Pairwise paired t-test of SDI values between these segments with the others were all significantly different (P<0.001, adjusted for multiple testing). The mean temporal EF curves in Fig. 1 (right) also confirm this phenomenon. This had a large effect on the SDI measurement (Table 1). When these segments were excluded, SDI values from all groups were significantly different (P<0.05) compared to when all segments included. The average SDI values were all decreased and the variations within each group were all smaller when S2 and S3 were excluded.

**Table 1:** Mean ± standard deviation of SDI values from different groups.

Group	Size	All segments	No septal base	P-value
Asymptomatic	292	5.8 ± 2.5%	4.2 ± 1.8%	< 0.001
Hypertrophy	12	6.5 ± 4.9%	3.8 ± 2.7%	0.04
Heart Failure	24	11.7 ± 5.6% **	10.6 ± 5.5% **	0.007

Two measurements were performed on each group, one with all segments included and the other omitting the septal base (S2 and S3) segments. P-value column was paired t-test between two measurements. Unpaired t-test between a particular group and the asymptomatic group was performed and marked with ** for P<0.001.

## Conclusions

Late regional contraction in septal base has a significant effect on the SDI values. Since SDI is measured by the dispersion of time, this effect was dominant. The delayed, as well as the increased variability, time-to-peak regional EF in these segments both seem to contribute to SDI variability. This study shows that eliminating septal base segments significantly decreased SDI values and narrowed their variation. Further validation is still needed to investigate whether this elimination protocol will better predict CRT responses.

## Funding

1) The Cardiac Atlas Project, NIH - National Heart, Lung and Blood Institute (R01HL087773), and 2) Faculty Research Development Fund (FDRF), The University of Auckland.
